# Supplementary material for: Cardiopulmonary resuscitation in veno-venous-ECMO patients—A retrospective study on incidence, causes and outcome
Source: PLoS One. 2023 Aug 11;18(8):e0290083. doi: 10.1371/journal.pone.0290083 (PMC10420365; doi:10.1371/journal.pone.0290083)
Supplement: Attachment — Submitted filename: point_by_point_Revision_Journal Requirements.docx [file pone.0290083.s001.docx]

**Revision**

**Journal Requirements:**

When submitting your revision, we need you to address these additional requirements.

**1.** Please ensure that your manuscript meets PLOS ONE's style requirements, including those for file naming. The PLOS ONE style templates can be found at

<https://journals.plos.org/PLOSOne_formatting_sample_main_body.pdf>

and

<https://journals.plos.org/PLOSOne_formatting_sample_title_authors_affiliations.pdf>

We revised manuscript accordingly and it now meets PLOS ONE’s style requirements.

**2.** Thank you for stating the following in the Competing Interests section: "..."

Please confirm that this does not alter your adherence to all PLOS ONE policies on sharing data and materials, by including the following statement: ""This does not alter our adherence to  PLOS ONE policies on sharing data and materials.” (as detailed online in our guide for authors http://journals.plos.org/plosone/s/competing-interests).  If there are restrictions on sharing of data and/or materials, please state these. Please note that we cannot proceed with consideration of your article until this information has been declared.

Please include your updated Competing Interests statement in your cover letter; we will change the online submission form on your behalf.

Our cover letter now includes the statement above in which we declare that our disclosed interests do not alter our adherence to PLOS ONE policies on sharing data and materials.

**3.** We note that you have indicated that data from this study are available upon request. PLOS only allows data to be available upon request if there are legal or ethical restrictions on sharing data publicly. For more information on unacceptable data access restrictions, please see <http://journals.plos.org/plosone/s/data-availability#loc-unacceptable-data-access-restrictions>.

The original data contains potentially highly sensitive patient data, so that according to the local strict data protection jurisdiction, publication of the original data set is prohibited by the jurisdiction as well as the ethics committee.

We adjusted our data availability statement accordingly:

**“The dataset supporting the conclusions of this article contained potentially highly sensitive patient data, such that publication of the original dataset is prohibited by both the jurisdiction and the Ethics Committee under strict local data protection jurisprudence. Data disclosure may be addressed to the corresponding author (ANF)** [**armin.flinspach@kgu.de**](mailto:armin.flinspach@kgu.de) **or the Data Protection Representative** [**datenschutz@kgu.de**](mailto:datenschutz@kgu.de) **upon reasonable request.”**

**4.** Your ethics statement should only appear in the Methods section of your manuscript. If your ethics statement is written in any section besides the Methods, please delete it from any other section.

We deleted the ethics declaration at the bottom of our manuscript. It is only found in the methods section now.

**5.** We note that Figure 2 in your submission contain copyrighted image. All PLOS content is published under the Creative Commons Attribution License (CC BY 4.0), which means that the manuscript, images, and Supporting Information files will be freely available online, and any third party is permitted to access, download, copy, distribute, and use these materials in any way, even commercially, with proper attribution. For more information, see our copyright guidelines: <http://journals.plos.org/plosone/s/licenses-and-copyright>.

We require you to either (1) present written permission from the copyright holder to publish these figures specifically under the CC BY 4.0 license, or (2) remove the figures from your submission:

We would like to apologize to the Editorial Office for the confusion. The graphic was created independently by the Corresponding Author itself using the freely available vector graphics program Inkscape version 1.2.2. Accordingly, the copyright is the exclusive property of the author. If the publisher has any concerns about similarities between the image and other published graphics, please let us know so that we can make appropriate adjustments to our graphics.
